# Early Detection of Pathological Gambling: Betting on GPs' Beliefs and Attitudes

**DOI:** 10.1155/2014/360585

**Published:** 2014-08-27

**Authors:** Sophia Achab, Anne Chatton, Riaz Khan, Gabriel Thorens, Louise Penzenstadler, Daniele Zullino, Yasser Khazaal

**Affiliations:** Addiction Division, Department of Mental Health and Psychiatry, University Hospitals of Geneva, 1202 Geneva, Switzerland

## Abstract

Pathological gambling (PG) is an addictive disorder with harm related to the high psychiatric comorbidity and increased suicidal risk. Prevalence rates in general population range from 0.2% to 2.1%. Problem gamblers are hard to attract to treatment programs for several proper reasons and for obstacles (e.g., accessibility). To address these obstacles, primary care (where the problem gambling (PrG) prevalence seems to be 6.2%) has a crucial role to play (i.e., identifying and referring patients to specialized treatment programs and treating at first line when needed and possible) in the era of online gambling offer expansion. The present work aimed to collect data on resources in the field from GPs themselves, using a 24-item online questionnaire. Swiss French-speaking participants were asked about their screening practice and knowledge. The results state that the vast majority of them are aware of the existence and the potential impact of PrG on their patients. However, PrG screening is not systematic and their knowledge of adequate treatments or referral methods is scarce. GPs being central to health screening in general, targeted advice and training on short screening tools and better knowledge of referral pathways should be promoted and continued to empower the GP's management skills in a public health approach.

## 1. Introduction

Pathological gambling (PG) has been recently added as a gambling disorder to the substance-related disorders chapter of DSM 5 [1], as a result of the empiric findings provided by the research literature supporting its similarity with substance use disorders (SUD). Indeed, it has been shown that PG shares clinical expression, comorbidity, neurobiological mechanisms [2–5], and treatment options [6–8] with SUD and reward-related behaviors.

PG harm related to its high psychiatric comorbidity, mostly substance use disorders [9] and increased suicidal risk [10]. Vulnerable subgroup populations such as adolescents are also affected by gambling disorders [11]. Prevalence rates in the general population range are ranging from 0.2% to 2.1% [12–14] for pathological gamblers and from 0.6% to 5.5% for problem gamblers [13, 15–18]. The prevalence seems to be more important (6.2%) in primary care services [19]. Problem gamblers are hard to attract to treatment programs, partly due to their feelings of shame and denial [20]. Only 0.4% to 3% of them seek help for their difficulties [21, 22] and a five-year latent period is observed between the first symptomatic presentation and the first attempt to seek care [23]. Hence, general practitioners (GPs) as primary care providers have a crucial role to play in the early detection and intervention on problem gambling (PrG) [24, 25]. There is a paucity of studies on the PrG management resources and screening practices of GPs. Fourteen years ago, in Canada, a structured national plan was designed to evolve physicians in PrG management [26]. The needs (PrG resources available and awareness on their existence) were studied in a sample of 54 physicians from the 800 contacted. Results showed a low awareness on PrG resources that have been considered by participants insufficient to fulfill the needs [26]. Concern about the lack of knowledge, education, and training in PrG and its perception as a nonmedical problem but rather as a character defect was raised as challenges and obstacles to GPs' evolvement in PrG management [26]. An Australian paper [24] presented the way GPs can help in early detection and intervention and reported a pilot project that provided resources to GPs. Results from the 24 GPs (with referral experience in PrG) from the 51 that received information and material on PrG (e.g., importance, list of referral services, and simple advice on the way to assist patients). The majority of participants were convinced of the role they can play in PrG management [24]. However, lack of knowledge was reported by almost half of the sample (even if they had referral experience in the field) and a difficulty to ask patients “out of the blue” if they gamble [24].

Another awareness study of PrG in 180 health care providers (nurses, physicians, and social workers) [27] showed that the vast majority are aware of the existence of PrG but only a minority are effectively screening their patients.

Screening for health problems in care providers themselves is not a frequent question. Regarding PG, a prevalence rate in American general practitioners of 5% has been reported [28].

This study aims first to evaluate interest and knowledge of GPs regarding PrG and the way they deal with it in their daily clinical practice. Secondly, it aims to screen for PrG in GPs themselves.

## 2. Methods

### 2.1. Sample

Swiss GPs with a medical practice in the 6 French-speaking areas (FSAs) of Switzerland were invited to participate anonymously in an online survey.

Participants were recruited between March and May 2011 via their physician's regional association through an e-mail informing about the study's aims. The participants were directed through a web link to the questionnaire.

### 2.2. Measures

A 24-item online questionnaire was developed for the study on Survey Monkey software. After sociodemographic data (Table 1), five items investigated participants' beliefs on PrG (Table 2). Then, participants were asked about their PrG screening practice (Table 3). They were presented a text-response item (to avoid oriented responses) to specify the PrG screening tools they use. They were also invited to estimate the rate of PrG and related debts issues in their active pool of patients. Practitioners were then asked how they manage PrG and its financial consequences in their patients (Table 3). The last section of the questionnaire consisted of items on the participants' impression about their knowledge of PrG disorder, on the existing specialized local treatment network, and their estimated need for information and training (Table 4). At the end of the questionnaire, responders were themselves screened for PrG, using the 2-item Lie-bet test [29] “*Have you ever felt the need to bet more and more money?”* and* “Have you ever had to lie to people important to you about how much you gambled?”* (Table 5).

### 2.3. Statistical Analysis

SPSS 18.0 (Statistical Package for the Social Sciences, IBM Inc., Chicago) software program was used to perform the statistical analyses. First, descriptive statistics were computed for the participants' characteristics (demographics and beliefs representation) and reported as medians, ranges, and percentages. For the sake of completeness, missing data are also provided in the tables.

Next, we looked for associations between screening frequency and GPs' interest in PrG, between knowledge of PrG, respectively, knowledge of PrG network, and screening practice for PrG, and finally between the need for information/training on PrG and knowledge of the topic, using the Pearson chi-square tests. When the expected frequency criteria were not met due to small cell sample size, adjacent categories were collapsed into smaller categories, where appropriate, in order to fulfill the necessary Pearson chi-square requirements and to gain statistical power. Two-by-two tables that did not meet these requirements were analyzed by the Fisher exact tests. Hence, for instance, knowledge of the topic reduces to two categories: very satisfactory/satisfactory versus insufficient/no knowledge. The same is the case for screening for excessive gambling frequency (systematically/often versus rarely/never) and demand for more information and training (total agreement/partial agreement versus partial disagreement/total disagreement).

## 3. Results and Discussion

The survey was relatively well received by Swiss GPs' professional associations in the French speaking area with 66% of acceptance to relay the information and the link to the online questionnaire. The sample consisted of 71 GPs accepting to participate in the survey. A majority of them (95.8%) filled out the questionnaires. Respondents were mostly men (63.2%), with a median age of 53 years and a median practice experience of 17 years as GP (Table 1). The vast majority is qualified specialists in primary care (general practitioner and/or internist) and their area of practice is given in Table 1.

When GPs were asked to estimate PrG rate in their active pool of patients, more than half of them did not answer and 11% declared not knowing this rate, while 24% of them estimated this rate (between 1 and 30%).

### 3.1. GPs' Beliefs on PrG and Financial Debts

The great majority (99%) expressly recognized believing in the potential addictive properties of gambling and 69% of them showed a keen interest in PrG with all the subsequent financial harm (Table 2). Two-thirds of them (62%) characterized PrG as an important or very important issue of concern in the French-speaking area of Switzerland. The whole sample agreed that gambling could lead to indebtedness and 89% agreed with the worsening of indebtedness related to excessive gambling.

### 3.2. GPs' Attitudes towards PrG

In their daily practice, while debts were often or systematically screened by 35% of the practitioners, PrG was screened only by a minority (7%) of them (Table 3). Screening habits were during general history taking or PrG being discovered by chance with the occurrence of payment difficulties. There was no relationship found between screening frequency and GPs interest in it (*P* = 1). Investigating PrG management, 52% of GPs referred their patients to a specialist and 7% treated it themselves, while 32% stated they do not know what to do with these problematic patients and 3% do not address this issue at all (Table 3). GPs promote a specialized approach to PrG treatment, in multidisciplinary centers (80%) and by private psychiatrists (3%). In debt management, GPs seemed to be more active than for PrG, with a greater rate of them treating it themselves (21%) and a lesser rate of “I do not know” (10%) responses.

### 3.3. Self-Reported Knowledge of PrG

Participants estimated their knowledge of PrG and on specialized care network as being null (resp., 14% and 25%) or unsatisfying (resp., 65% and 45%) (Table 4). This was found to be independent of their screening practice for problem gambling (resp., *P* = 0.2 and *P* = 0.1). The majority of participants reported a need for information (86%) and for training (77.5%) on PrG (Table 4). This need was found to be independent of how satisfied they felt about their feeling as satisfied or not from their knowledge of the topic (*P* = 0.5).

One participant screened himself positive for problem gambling according to Lie-bet items [29].

In summary, data showed that the majority of GPs considered gambling addictive and they believed in the importance of problem gambling in their area of practice, estimating furthermore a high rate of PrG and related indebtedness in their own patients. These results are different from those of the Canadian sample of physicians in 2000 [26] but similar to those from the Australian data in 2007 [24]. This highlights the possible mentality changes this last decade regarding PrG status as a medical disorder and constitutes a better chance for GPs to be motivated to play a role in its management. Nevertheless, screening practice was very low and PrG was often discovered by chance when patients experienced financial issues. In addition, GPs interested in PrG did not differ significantly in screening from those who declared less or no interest in the field. This could be explained by the gap between beliefs and attitudes in a real practice setting. Even if GPs believe and take interest in PrG, they probably tend to prioritize managing other disorders (i.e., somatic and/or with short- or medium-term vital risk). They could also feel a lack of time in their consultation to include questions on PrG [30]. This goes in line with the obstacles stated in recent literature to be facing GPs' evolvement in PrG screening (e.g., “lack of time” and “PrG considered as a new problem having a low incidence”) [26]. GPs could interest in PrG but could lack suitable and available resources and knowledge on PrG care management. The economically symptomatic PrG (i.e., patient declaring financial issues or incidents of fee payment issues) could be a sign of alert of the disorder for the practitioner, but unfortunately financial consequences are already present. This aspect could be addressed by renewed information on the vital risk of PrG (e.g., suicidal risk) and the importance of the early detection. GPs should also be trained and continuously trained to use basic and suitable PrG screening tools to detect patients before crisis-driven help seeking. GPs in the present work experienced to be screened for PrG using the Lie-bet items. This could have led to an awareness of an existing short and easy screening tool they can use in their daily practice.

Another contrast between GPs beliefs and attitudes regarding PrG is that even if the majority of GPs knew the best treatment approach as being multidisciplinary, only half of them referred to these kinds of treatment systems. The poor knowledge reported on the specialized local treatment network could explain these findings. This aspect could be addressed by a wider dissemination, through GPs professional associations, of the current accessible information about PrG local treatment systems. Internet could be an interesting, fast, cost-effective, and easy-to-use vector for such information and training dissemination. Several countries have specific web-based information on PrG including information on the local and national specialized treatment centers (i.e., http://www.sos-jeu.ch/, http://www.jeu-aidereference.qc.ca/, and http://www.problemgamblingguide.com/). One possible intervention by GPs once patients screened could be a brief counseling consisting in the recommendation to their patients to visit such web pages to get information on the disorder and the specialized ways of help they could seek. Several medical associations have developed specific material targeting GPs to help them inform their patients on gambling and how to manage PrG in general practice [24]. Since problem gamblers seem to be more likely to accept help from their general practitioner regarding this disorder [31], pharmacotherapy for PrG [6–8] could be an interesting option as it fits with a general practice setting.

A large number of participants stated themselves (79%) as dissatisfied with their knowledge of the disorder and the referring structures and the large majority of the sample declared needing more information (86%) and training (77.5%) on PrG and its management. This is a need that should be addressed by structured specific training and support strategies. Helplines for GPs and supervisions should be considered in addition to specifically designed training materials and settings (i.e., pregraduate, postgraduate, and continuous training). E-learning and distance supervisions (e.g., through e-mails or videoconferences) are emerging tools to build capacity that demonstrated efficacy in other fields in medicine web-platforms dedicated to map and to inform professionals on the tendencies on some addictive behaviors are currently developing [32–35].

The high rate of missing data concerned electively the second part of the questionnaire based on attitudes and knowledge. Taking into account that most of the participants answered to the beliefs, this could be explained by social desirability (i.e., difficulty to report the ignorance on a topic).

With the lack of information on the rate of participants from the panel sought (unknown proportion of affiliated doctors in each professional association at the time of the study), the representativeness of the sample here studied is hard to describe. Furthermore, the only data available is the number of 1183 of Swiss doctors (including GPs) in outpatient sector of the geographic areas concerned by our survey [26, 27, 36]. Another limitation of this work is the predictable lack of statistical significance in the associations testing between beliefs and attitudes due to the small sample size and the missing data. However, descriptive data is the most important contribution of our work. Validity of our results can be appreciated by some indirect indicators. Firstly, data on GPs' attitudes of PrG screening and knowledge are in line with previous studies [24]. Secondly, the proportion of probable PrG in the sample itself (1.5%) was situated in the range of the general Swiss population prevalence [15, 17]. Finally, even if the sample is moderate, a wide age range (34–70 years old) of GPs was represented. Participants, having done their medical studies at different periods in time, represent the panel of different considerations of the PrG as a disorder for the medical community in the last decades.

To our knowledge, this is the first study specifically targeting GPs (regardless to their PrG referral experience) to investigate their beliefs, resources, and practice related to PrG, above all, in the era of an expanding offer of online gambling.

## 4. Conclusion

The results state that the vast majority of Swiss GPs that participated in the study are aware of the existence and the potential impact of PrG on their patients. But, as expected, the screening of PrG is not systematic and their knowledge of adequate treatments or referral methods is scarce. The discrepancy between beliefs in the harm related to PrG and the lack of its management could be addressed by information, training, and support for general practitioner. The implementation and success of such plan will be facilitated as GPs specifically stated this need. GPs being central to health screening in general and the pressure on them to screen almost all health issues, targeted advice and training (e.g., short screening tools, better knowledge of when to refer to a specialist, and effective pharmacotherapy strategies) should be promoted to empower the GP's management skills in the context of a public health approach. This training and information should be periodically renewed to face new challenges (e.g., Internet as a vector of gambling accessibility but also information and training vector) and to know new management strategies. Our findings can be the first stepping stone in the implementation of such capacity building strategy for PrG early detection and intervention according to the local context. Indeed, concrete tracks can be designed starting from this inventory of representations, knowledge, practice habits, and needs. Such strategy could be inspired by previous afterthoughts [24–26]. This study may have served as a brief intervention to remind the existence and the harms of this disorder. Screening problematic gambling in GPs themselves could have been a novel way to make them aware of possible simple and fast screening tools. The goal of enabling general practitioners is to improve the early detection of problem gamblers and to increase their treatment seeking.

## Figures and Tables

**Table 1:** Sociodemographic data.

Total sample (*N* = 71)	
Age (years), median (min–max)	53 (34–71)
Gender, *n* (%)	
Female	25 (35.2)
Male	43 (60.6)
Missing	3 (4.2)
Practice duration (years), median (min–max)	17 (1–38)
Medical specialization, *n* (%)	
General practitioner	33 (46.5)
Internist	33 (46.5)
General practitioner and internist	1 (1.4)
Internist and other	3 (4.2)
No specialization	1 (1.4)
Area of practice, *n* (%)	
Fribourg	1 (1.4)
Geneva	31 (43.7)
Jura	0 (0)
Neuchâtel	23 (32.4)
Valais	0 (0)
Vaud	16 (22.5)

**Table 2:** Participants beliefs on excessive gambling.

Total sample (*N* = 71)	*n* (%)
In your opinion, excessive gambling in Swiss French-speaking area is	
Not an issue	0 (0)
A minor issue	18 (25.4)
A major issue	41 (57.7)
A very major issue	3 (4.2)
I do not know	9 (12.7)
Your interest in excessive gambling and gamblers' indebtedness is	
Important	11 (15.5)
Medium	38 (53.5)
Low	18 (25.4)
Null	2 (2.8)
I do not know	2 (2.8)
Do you think gambling could become excessive or addictive	
Total agreement	66 (93.0)
Partial agreement	4 (5.6)
Partial disagreement	0 (0)
Total disagreement	0 (0)
I do not know	1 (1.4)
Do you think gambling could lead to indebtedness	
Total agreement	69 (97.2)
Partial agreement	2 (2.8)
Partial disagreement	0 (0)
Total disagreement	0 (0)
I do not know	0 (0)
Does excessive gambling worsen indebtedness in the current economical context	
Total agreement	45 (63.4)
Partial agreement	18 (25.4)
Partial disagreement	2 (2.8)
Total disagreement	0 (0)
I do not know	5 (7.0)
Missing	1 (1.4)

**Table 3:** Participants attitudes towards excessive gambling.

Total sample (*N* = 71)	*n* (%)
Do you screen for excessive gambling	
Systematically	0 (0)
Often	5 (7.0)
Rarely	25 (35.2)
Never	22 (31.1)
I do not know	1 (1.4)
Missing	18 (25.4)
Do you screen for indebtedness	
Systematically	1 (1.4)
Often	24 (33.8)
Rarely	24 (33.8)
Never	6 (8.5)
I do not know	2 (2.8)
Missing	14 (19.7)
Your attitude towards excessive gambling is	
I refer to specialist	37 (52.1)
I treat it	5 (7.0)
I do not do anything	2 (2.8)
I do not know	22 (31.8)
Missing	5 (7.0)
Your attitude towards indebtedness is	
I refer to specialist	34 (47.9)
I treat it	15 (21.1)
I do not do nothing	3 (4.2)
I do not know	7 (9.9)
Missing	12 (16.9)
The best management of excessive gamblers is in referral to	
Specialized multidisciplinary centers (doctors, psychologists, and social workers)	57 (80.3)
Private psychiatrists	2 (2.8)
General practitioners	3 (4.2)
Social services	1 (1.4)
Other	3 (4.2)
I do not know	2 (2.8)
Missing	3 (4.2)

**Table 4:** Self-reported knowledge of problem gambling.

Total sample (*N* = 71)	*n* (%)
My knowledge of problem gambling is	
Very satisfying	0 (0)
Satisfying	12 (16.9)
Dissatisfying	46 (64.8)
Null	10 (14.1)
I do not know	0 (0)
Missing	3 (4.2)
My knowledge of problem gambling care network is	
Very satisfying	0 (0)
Satisfying	15 (21.1)
Dissatisfying	32 (45.1)
Null	18 (25.4)
I do not know	3 (4.2)
Missing	3 (4.2)
I desire more information about problem gambling	
Total agreement	39 (54.9)
Partial agreement	22 (31.0)
Partial disagreement	3 (4.2)
Total disagreement	2 (2.8)
I do not know	1 (1.4)
Missing	4 (5.6)
I desire more training on problem gambling	
Total agreement	19 (26.8)
Partial agreement	36 (50.7)
Partial disagreement	6 (8.5)
Total disagreement	3 (4.2)
I do not know	1 (1.4)
Missing	6 (8.5)

**Table 5:** Screening for PrG in participants.

Total sample (*N* = 71)	*n* (%)
Have you ever felt the need to bet more and more money	
Yes	1 (1.4)
No	67 (94.4)
I do not know	0 (0)
Missing	3 (4.2)
Have you ever had to lie to people important to you about how much you gambled	
Yes	0 (0)
No	68 (95.8)
I do not know	0 (0)
Missing	3 (4.2)

## Abbreviations

FSAs: French-speaking areas
GPs: General practitioners
PG: Pathological gambling
PrG: Problem gambling.

## References

[1] APA http://www.dsm5.org/Pages/Default.aspx.

[2] Petry NM, Blanco C, Auriacombe M (2014). An overview of and rationale for changes proposed for pathological gambling in DSM-5. *Journal of Gambling Studies*.

[3] Baik JH (2013). Dopamine signaling in food addiction: role of dopamine D2 receptors. *BMB Reports*.

[4] Angelucci F, Martinotti G, Gelfo F (2013). Enhanced BDNF serum levels in patients with severe pathological gambling. *Addiction Biology*.

[5] Bowden-Jones H, Clark L (2011). Pathological gambling: a neurobiological and clinical update. *British Journal of Psychiatry*.

[6] Achab S, Khazaal Y (2011). Psychopharmacological treatment in pathological gambling: a critical review. *Current Pharmaceutical Design*.

[7] Martinotti G, Lupi M, Sarchione F (2013). The potential of pregabalin in neurology, psychiatry and addiction: a qualitative overview. *Current Pharmaceutical Design*.

[8] Pettorruso M, Martinotti G, di Nicola M (2012). Amantadine in the treatment of pathological gambling: a case report. *Frontiers in Psychiatry*.

[9] Bischof A, Meyer C, Bischof G, Kastirke N, John U, Rumpf H (2013). Comorbid Axis I-disorders among subjects with pathological, problem, or at-risk gambling recruited from the general population in Germany: results of the PAGE study. *Psychiatry Research*.

[10] Thon N, Preuss UW, Pölzleitner A (2014). Prevalence of suicide attempts in pathological gamblers in a nationwide Austrian treatment sample. *General Hospital Psychiatry*.

[11] Villella C, Martinotti G, Di Nicola M (2011). Behavioural addictions in adolescents and young adults: results from a prevalence study. *Journal of Gambling Studies*.

[12] Commission AGP (1999). *Australia’s Gambling Industries*.

[13] Jonsson J, Psykologi P (2006). An overview of prevalence surveys of problem and pathological gambling in the Nordic countries. *Journal of Gambling Issues*.

[14] Meyer G, Hayer T, Griffiths M (2009). *Problem Gambling in Europe: Challenges, Prevention, and Interventions*.

[15] Bondolfi G, Osiek C, Ferrero F (2000). Prevalence estimates of pathological gambling in Switzerland. *Acta Psychiatrica Scandinavica*.

[16] Welte J, Barnes G, Wieczorek W, Tidwell M-C, Parker J (2001). Alcohol and gambling pathology among U.S. adults: prevalence, demographic patterns and comorbidity. *Journal of Studies on Alcohol*.

[17] Bondolfi G, Jermann F, Ferrero F, Zullino D, Osiek CH (2008). Prevalence of pathological gambling in Switzerland after the opening of casinos and the introduction of new preventive legislation. *Acta Psychiatrica Scandinavica*.

[18] Kairouz S, Nadeau L, Paradis C (2011). Portrait du jeu au Québec: prévalence, incidence et trajectoires sur quatre ans. *Enquête ENHJEU- Québec*.

[19] Pasternak AV (1999). Prevalence of gambling disorders in a primary care setting. *Archives of Family Medicine*.

[20] Suurvali H, Cordingley J, Hodgins DC, Cunningham J (2009). Barriers to seeking help for gambling problems: a review of the empirical literature. *Journal of Gambling Studies*.

[21] Evans L, Delfabbro PH (2005). Motivators for change and barriers to help-seeking in australian problem gamblers. *Journal of Gambling Studies*.

[22] Suurvali H, Hodgins D, Toneatto T, Cunningham J (2008). Treatment seeking among Ontario problem gamblers: results of a population survey. *Psychiatric Services*.

[23] Tavares H, Zilberman ML, Beites FJ, Gentil V (2001). Gender differences in gambling progression. *Journal of Gambling Studies*.

[24] Tolchard B, Thomas L, Battersby M (2007). GPs and problem gambling: can they help with identification and early intervention?. *Journal of Gambling Studies*.

[25] Sanju G, Gerada C (2011). Problem gamblers in primary care: can GPs do more?. *The British Journal of General Practice*.

[26] Rowan MS, Galasso CS (2000). Identifying office resource needs of Canadian physicians to help prevent, assess and treat patients with substance use and pathological gambling disorders. *Journal of Addictive Diseases*.

[27] Christensen MH, Patsdaughter CA, Babington LM (2001). Health care providers' experiences with problem gamblers. *Journal of Gambling Studies*.

[28] Bazargan M, Makar M, Bazargan-Hejazi S, Ani C, Wolf KE (2009). Preventive, lifestyle, and personal health behaviors among physicians. *Academic Psychiatry*.

[29] Johnson EE, Hamer R, Nora RM, Tan B, Eisenstein N, Engelhart C (1997). The lie/bet questionnaire for screening pathological gamblers. *Psychological Reports*.

[30] McCambridge J, Cunningham J (2007). Against the odds: should GPS have any involvement with gambling problems?. *British Journal of General Practice*.

[31] Sullivan S, McCormick R, Lamont M, Penfold A (2007). Problem gambling: patients affected by their own or another's gambling may approve of help from general practitioners. *New Zealand Medical Journal*.

[32] Clarke A, Lewis D, Cole I, Ringrose L (2005). A strategic approach to developing e-learning capability for healthcare. *Health Information and Libraries Journal*.

[33] Ritchie A (2011). The Library's role and challenges in implementing an elearning strategy: a case study from northern Australia. *Health Information & Libraries Journal*.

[34] Hackett J, Madden DL, Viney KA, Naylor C (2009). Evaluation of three population health capacity building projects delivered by videoconferencing in NSW. *New South Wales Public Health Bulletin*.

[35] Corazza O, Assi S, Simonato P (2013). Promoting innovation and excellence to face the rapid diffusion of novel Psychoactive substances in the EU: the outcomes of the reDNet project. *Human Psychopharmacology*.

[36] Confédération suisse O.f.d.l.s http://www.bfs.admin.ch/bfs/portal/fr/index/themen/14/03/03/key/01.html.

